# A Holistic View of Berberine Inhibiting Intestinal Carcinogenesis in Conventional Mice Based on Microbiome-Metabolomics Analysis

**DOI:** 10.3389/fimmu.2020.588079

**Published:** 2020-09-24

**Authors:** Haitao Chen, Fan Zhang, Jin Zhang, Xinjie Zhang, Yong Guo, Qinghua Yao

**Affiliations:** ^1^The First Clinical Medical College, Zhejiang Chinese Medical University, Hangzhou, China; ^2^The Second Clinical Medical College, Zhejiang Chinese Medical University, Hangzhou, China; ^3^Department of Oncology, The First Affiliated Hospital of Zhejiang Chinese Medical University, Hangzhou, China; ^4^Department of Integrated Traditional Chinese and Western Medicine, Cancer Hospital of the University of Chinese Academy of Sciences (Zhejiang Cancer Hospital), Hangzhou, China

**Keywords:** berberine, colorectal cancer, gut microbiota, NMR-based metabolomics, metabolites

## Abstract

Berberine (BBR) has been reported that it has effects on inhibiting colorectal cancer (CRC). However, the mechanism of BBR on CRC also remains largely unknown. Herein, we investigated the therapeutic effects of BBR on CRC from the perspective of gut microbiota and metabolic alterations, which can provide a holistic view to understand the effects of BBR on CRC. First, azoxymethane (AOM)/dextran sodium sulfate (DSS) mouse was used as CRC animal model, then the degree of colorectal carcinogenesis in AOM/DSS mice with or without BBR administration was measured. The composition and abundance of gut microbiota was investigated by using 16S rRNA. Meanwhile, feces samples were analyzed with ^1^H NMR spectroscopy to investigate the metabolic alterations. As a result, BBR significantly reduced intestinal tumor development with lower macroscopic polyps and ki-67 expression of intestinal tissue, and better colonic morphology in mice. Moreover, BBR altered the composition of gut microbiota in AOM/DSS mice obviously, which were characterized by a decrease of *Actinobacteria* and *Verrucomicrobia* significantly at the phylum level. At the genus level, it was able to suppress pathogenic species, such as *f_Erysipelotrichaceae*, *Alistipes*, and elevate some short-chain fatty acids (SCFA)-producing bacteria, including *Alloprevotella*, *Flavonifractor*, and *Oscillibacter*. Metabolic data further revealed that BBR induced metabolic changes in feces focus on regulating glycometabolism, SCFA metabolism and amino acid metabolism, which also provides evidence for alteration of the microbiota because these feces metabolites are the products of interactions between the host and the microbial community. This study showed that BBR induced alterations in microbiota and metabolic in AOM/DSS mice, which might providing new insight into the inhibition effects of BBR on CRC.

## Introduction

Berberine (BBR) is a natural pentacyclic isoquinoline alkaloid, which is isolated from Chinese herbs, such as *Coptis chinensis, Hydrastis, Cortex Phellode*, and *Berberis* (1). Multiple studies have confirmed that BBR had a variety of bioactivities, including anti-inflammatory (2, 3), anti-microbial (4), and hypoglycemic and lipid-lowing efficacy (5, 6). Several clinical and preclinical studies also demonstrate the ameliorative effect of berberine against several disorders including metabolic, neurological, cardiological, and gastrointestinal problems (7, 8). Additionally, 9 conducted a double-blind, randomized, placebo-controlled clinical trial, and found that BBR at 0.3 g twice daily was safe and effective in reducing the risk of recurrence of colorectal adenoma (9). Therefore, BBR is currently gaining more attention due to its promising therapeutic effects on colorectal cancer (CRC). Moreover, emerging evidence demonstrated that BBR suppressed colon tumorigenesis via controlling cell signaling pathways, inducing apoptosis, attenuating oxidative stress, and inhibiting inflammatory response (10). However, its clinical efficacy also hard to be explained entirely because its absolute bioavailability is very low (11). Thus, the pharmacological mechanism of BBR on CRC needs further research.

Colorectal cancer is a leading cause of cancer-related mortality in the world (12). It is well known that CRC is a multifactorial disorder involving both genetics (13, 14) and external environment (such as diet, smoking, and lifestyle) (14–16). Recently, an increasing number of studies suggested that gut microbiota played an important role in intestinal disorders, especially including CRC (17–19). With the developing of microbiome technology, the dysbiosis in CRC has been further found to involve in the decrease of some beneficial bacteria, such as *Lactobacillus*, *Eubacterium rectale* (20), and increase of pathogenic bacteria, such as *Fusobacterium nucleatum*, *Escherichia coli* (21). In addition, further studies indicated that the gut microbiota with its metabolites has been shown to be essential in the host metabolism of substances such as short-chain fatty acids (SCFAs) (22), which can directly inhibit the pathological progression of CRC (23, 24). Hence, the therapeutic strategies for CRC have now shifted to the microbiome-metabolomics. Interestingly, BBR has been reported to regulate the gut microbiota diversity and community composition, which contributed to attenuate obesity and insulin resistance, as well as CRC in high-fat diet-fed rats (25–27), suggesting that BBR can treat diseases by modulating gut microbiota. Here, we hypothesized that the gut microbiota and its metabolites are the targets of BBR on the treatment of CRC.

At present, according to the microbiota combined with metabolomics analysis, the interactions between the host and its microbiota can be elaborated systematically. Therefore, we used 16S rRNA gene sequencing to detect the alterations of microbiota and ^1^H NMR analysis to filtrate the differential metabolites in order to illuminate the mechanism of BBR on the treatment of CRC. This study aimed to identify the key bacteria and metabolites in BBR treated mice with CRC, which hoping to provide a new insight for BBR on CRC.

## Materials and Methods

### Animals and Experimental Design

**FIGURE 1:**
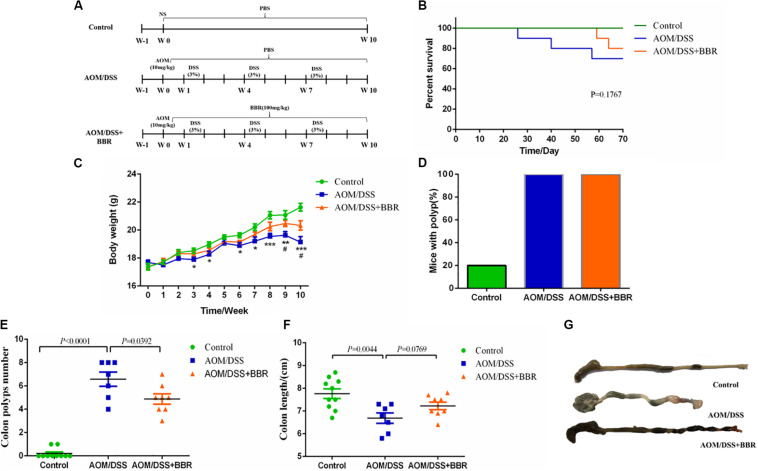
Effects of BBR on intestinal tumorigenesis in the AOM/DSS mouse model. **(A)** Design of BBR experiment to AOM/DSS mice (*n* = 10/group). **(B)** Survival rate of each group was measured. **(C)** Body weight of mice was recorded. **(D)** The rate of mice with polyp was detected. **(E)** Number of colon tumors was observed. **(F,G)** The colon length was measured. Data are expressed as mean ± SE. AOM/DSS (vs. Control: *P < 0.05, **P < 0.01, ***P < 0.001; vs. AOM/DSS + BBR: ^#^*P* < 0.05).

Specific pathogen free conventional female C57BL/6 mice (18–20 g) were purchased from Shanghai B&K Co., Ltd. (Shanghai, China). All mice were housed in the Laboratory Animal Center of Zhejiang Chinese Medical University with a controlled environment (temperature, 24 ± 2°C; humidity, 55 ± 10%; light, 12 h light/dark cycle). After 1 week of adaptive feeding, 30 mice were randomly divided into three groups (*n* = 10 in each group) according to the body weight: control group, azoxymethane (AOM)/dextran sodium sulfate (DSS) group, and AOM/DSS + BBR group. Referenced to the previous publication (28), AOM/DSS group and AOM/DSS + BBR group mice were injected with 10 mg/kg AOM (Sigma, United States) intra-peritoneally for one time; after 1 week, the mice began to free drink with 3% DSS (Beijing Solarbio Science & Technology Co., Ltd., China) for three cycles (given 3% DSS in drinking water for 1 week, and then given drinking water for 2 weeks). The control group was injected with one dose of 0.9% saline intra-peritoneally, and given drinking water all the time. Meanwhile, AOM/DSS + BBR group mice were treated with BBR (100 mg/kg; Shanghai Yuanye Bio-Technology Co., Ltd., China) by oral gavage once daily for 10 weeks. The dosage of BBR was referenced to the previous publication (29). Control group and AOM/DSS group were gavaged with drinking water (Figure 1A). The body weights were recorded once weekly.

### Sample Collection

At the end of the experiments, all mice were placed into the metabolic cages separately and the stool were collected and stored at −80°C immediately for further analysis. Then, all mice were fasted for 12 h and sacrificed on the last day of 12 weeks. The colon of each mice was excised and quickly washed with pre-cooled 0.9% saline. The length of colon was measured, and the number of polyps was recorded. Then, the colon was divided into two parts. One part was snap frozen in liquid nitrogen and then stored at −80°C for metabolic analysis, and the other was fixed in 10% neutralized formalin for histological examination. All tissue samples were kept on ice for as long as possible during the experiments.

### Histological Evaluation

Colon tissue was fixed in 10% neutralized formalin overnight and stored in 70% ethanol. Then the fixed sections of colonic tissues were embedded in paraffin, sectioned in 4 μm thick slices, and stained with hematoxylin and eosin (H&E) for histological analysis.

### Immunohistochemistry Staining

Paraffin-embedded colon tissues were used for analyzing the expression of Ki-67. After deparaffinized in citrate buffer solution (pH 6.0), the sections were pre-incubated in 3% H_2_O_2_ for 25 min and re-incubated in normal goat serum for 1 h. Sections were then incubated overnight in primary antibody solution dilute with anti-Ki 67 antibody (1:800; Abcam, United States) at 4°C. Slides were incubated with HRP-conjugated secondary antibody for 50 min. The sections were covered with DAB for several minutes, and counterstained with hematoxylin for 3 min. Finally, the sections were dehydrated with ethanol, sealed, and examined using image analysis software ImageProPlus 4.5. Representative images were captured from five independent samples.

### Sample Preparation for NMR Spectroscopy

The method of fecal sample preparation for metabolic profiling was operation according to the previous study (30). Briefly, 100 mg thawed stool material were mixed with 0.8 mL phosphate buffer saline (PBS) containing 10% deuterated water (D2O 99.8%; SIGMA, Untied States) and 0.05 mM sodium 3-trimethylsilyl-propionate-d4 (TMSP-2,2,3,3-d4; SIGMA, Untied States) as chemical shift reference. The mixture was kept on the ice for 30 min and then dissolved for 10 cycles (one cycle includes 20 s ultrasound, 10 s crash, and 30 s rest). Then the fecal slurry was centrifuged at 13,000 *g* for 10 min at 4°C for twice to obtained samples.

### Metabolites Analysis

The fecal and colorectal tissue samples were analyzed by ^1^H NMR spectroscopy analysis. The samples were transferred into 5 mm NMR tubes individually on a Bruker AVANCE III spectrometer equipped at 600 MHz with a 5 mm-BBFO probe. ^1^H NMR spectra were obtained by one dimensional NOESYPR1D pulse sequence. Free induction decays (FIDs) adopted a spectral width of 20 ppm with a mixing time of 100 ms and pulse delay time of 1.7 s, and they were collected with 128 transients into 32 k data points. An exponential function with a line-broadening factor of 0.3 Hz multiplied to all FIDs before Fourier transformation. The characteristic peaks of metabolites were detected according to the network database of metabolomics, including the Human Metabolome Database (HMDB^1^) and Biological Magnetic Resonance Bank (BMRB^2^).

### Pattern Recognition Analysis and Cross Validation

The principal components analysis (PCA) of the ^1^H NMR spectral data was made by SIMCA software, version 14.1. The orthogonal partial least squares-discriminant analysis (OPLS-DA) was applied to optimize the separation between different groups. The model quality was evaluated by the value of R2Y and Q2, which reflected the explained fraction of variance and the model predictability. The score of R2Y and Q2 closer to 1 demonstrates higher reliability of the prediction in the cross-validation procedure.

### DNA Extraction and 16S rRNA Gene Sequencing

Feces sample from four mice in each group were used for 16 rRNA gene sequencing. The total genomic DNA of gut microbiota was extracted from feces using the TIANamp Stool DNA Kit (DP328) (TIANGEN, China). Then, the concentration and integrity of DNA were measured by a microplate reader and agarose gel electrophoresis in advance of sequencing preparation. The V3–V4 regions of the 16S rRNA genes of the microbiota were amplified with bar-coded primers (Forward primer: 5′TCGTCGGCAGCGTCAGATGTGTATAAGAGACAGCCTAC GGGNGGCWGCAG; Reverse primer: 5′GTCTCGTGGGCT CGGAGATGTGTATAAGAGACAGGACTACHVGGGTATCTA ATCC) and sequenced using Illumina MiSeq platform. Subsequently, a mixture of PCR products was purified with the GeneJET Gel Extraction Kit (QIAGEN, Germany). After purification, the amplicons were equally combined and subjected to a sequencing library preparation according to the manufactory’s manual. The qualified libraries were sequenced on the HiSeq2500 platform (Illumina, United States). In addition, the sequences with 97% accordance among the remaining representative readings were assigned to the same operational taxonomic units (OTUs). We then assigned a taxonomy for each OTU representative sequence using the QIIME software package (Quantitative Insights Into Microbial Ecology).

### Statistical Analysis

All values were expressed as mean ± SE. The heatmap was made via Morpheus^3^. The differential metabolites were filtered by variable influence on projection (VIP) selection according to the PLS-DA and the filtering conditions VIP > 1 and *P* < 0.05. Differences between two groups were analyzed by un-paired Student’s *t*-test using GraphPad Prism 6.01. A two-tailed *P-*value of <0.05 was considered statistically significant.

## Results

### BBR Decreases Tumorigenesis in AOM/DSS Induced Colorectal Carcinogenesis

To determine the inhibitory effect of BBR on CRC progression, we gavaged BBR (100 mg/kg) to AOM/DSS induced colorectal carcinogenesis mice (Figure 1A). The survival curve indicated that the mortality of AOM/DSS group was higher than that of other groups (*P* = 0.1767, Figure 1B). In addition, the body weights of both AOM/DSS and AOM/DSS + BBR group mice were lower significantly compared to the control group (*P* < 0.05, Figure 1C). However, the body weight of the mice in AOM/DSS + BBR group was heavier than that in AOM/DSS group (*P* < 0.05, Figure 1C). Meanwhile, macroscopic examination showed that all mice had colonic polyps in AOM/DSS group (7/7 = 100%) and AOM/DSS + BBR group (8/8 = 100%), the rates were significantly higher than that of control group with two mice (2/10 = 20%) (Figure 1D). Furthermore, the number of polyps in AOM/DSS group was noticeably higher than that of control group (*P* < 0.01, Figure 1E). In contrast, BBR treatment significantly reduced the number of polyps in AOM/DSS mice (*P* < 0.05). Also, the length of colon in AOM/DSS and AOM/DSS + BBR group was significantly shorter than that in control group (*P* < 0.05, Figures 1F,G). Although there was no significant difference between AOM/DSS and AOM/DSS + BBR group, the average length of colon in AOM/DSS + BBR group was longer than AOM/DSS group.

**FIGURE 2:**
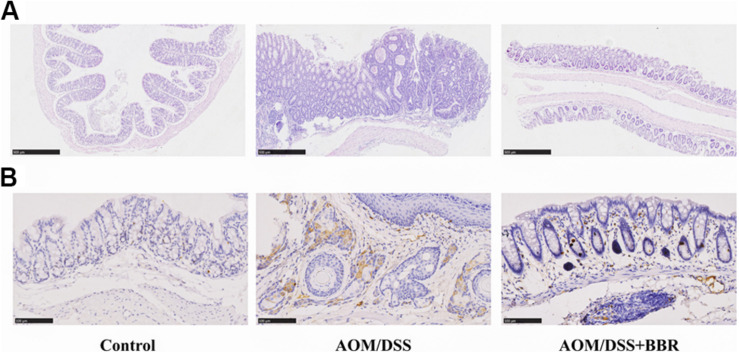
Effects of BBR on AOM/DSS induced colorectal carcinogenesis. **(A)** H&E staining of colorectal sections. **(B)** Immunohistochemistry staining of colorectal sections showing the expression of Ki-67 among groups.

The histological analysis showed that compared to the normal crypts from control group, the mice in AOM/DSS group had hypoplasia of crypt, hyperplasia of adenoma in mucosa with increased nucleus/cytoplasmic ratio (Figure 2A). Interestingly, these changes were greatly improved in AOM/DSS + BBR group. Furthermore, immunohistochemistry staining indicated that the expression of Ki-67 in colonic tissue was increased significantly in AMO/DSS group compared to the control group (Figure 2B), while their expressions in AOM/DSS + BBR group were remarkably reduced. Taken together, these data indicated that BBR decreased tumorigenesis in AOM/DSS induced colorectal carcinogenesis.

### BBR Regulated Imbalance of Gut Microbiota in AOM/DSS Mice

**FIGURE 3:**
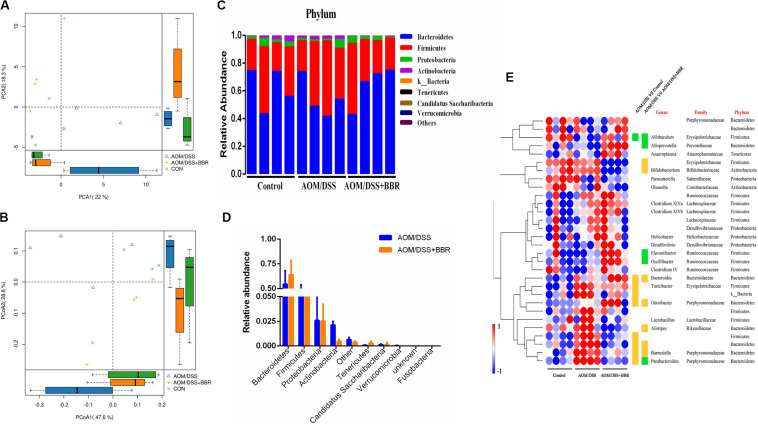
Diversity and composition of gut microbiota among groups (*n* = 4/group). **(A,B)** unsupervised PCA scatter plot and weighted uniFrac-based PCoA of feces microbiota. **(C)** Bacterial taxonomic profiling at the phylum level in different sample. **(D)** Relative abundances of bacterial phyla level between AOM/DSS group and AOM/DSS + BBR group, and the red marked bacteria have significant differences; **(E)** Heat map of the relative abundances of various bacterial genera identified in feces samples among groups. Each raw represents one genus with its family and phylum information, and blank means unclassified. Each column represents an individual sample. Red or blue color represents the high relative or low relative of abundance in each sample. Besides, green or orange entry indicates genera that were averagely less or more abundant in AOM/DSS group relative to control group, or to AOM/DSS + BBR group, and there were significant differences between the two groups (*P* < 0.05).

The changes of gut microbiota were revealed by using high throughput sequencing of the 16S rRNA gene. First, we examined the diversity and richness of microbiota through analyzing Chao1, observed species, Shannon index, and Simpson index. As shown in Table 1, no statistical difference was observed in the Shannon and Simpson indices among the three groups, but the Chao1 and observed species indices showed that the richness indices in the BBR treatment group were the lowest among the three groups (*P* < 0.01), indicating that BBR treatment greatly decreased the community richness of microbiota but did not affect the diversity of microbial community. In addition, unsupervised PCA scatter plot and weighted uniFrac-based PCoA analysis demonstrated that there was a significant difference among three groups according to the beta-diversity at OTUs level (Figures 3A,B). However, the distance between control group and AOM/DSS + BBR group was closer than that between control group and AOM/DSS group. These results indicated that BBR treatment could regulate the microbiota composition towards a similar proportion to the control group in AOM/DSS mice.

**TABLE 1:** Richness and diversity indices of gut microbiota among three groups.

**Group**	**Diversity indices**		**Richness indices**	
	**Shannon**	**Simpson**	**Chao 1**	**Observed OUTs**
Control	5.52 ± 0.34	0.94 ± 0.18	535.01 ± 51.20	403.50 ± 46.59
AOM/DSS	5.90 ± 0.25	0.96 ± 0.12	461.80 ± 11.65*	406.00 ± 13.96
AOM/DSS + BBR	5.34 ± 0.27	0.95 ± 0.12	393.05 ± 52.45**^ #^	302.25 ± 25.79*^ #^

The data are expressed as the mean ± SD (*n* = 4). AOM/DSS+BBR (vs. Control: *P < 0.05, **P < 0.01; vs. AOM/DSS: ^#^*P* < 0.05).

Further analysis, 10 phyla were recognized in the fecal microbiota by 16S rRNA analysis, and the details are showed in Figure 3C. AOM/DSS group had a relatively higher abundance of *Firmicutes* and lower *Bacteroidetes* than either control or AOM/DSS + BBR group. Furthermore, compared to the AOM/DSS group, BBR treatment decreased the relative abundance of *Actinobacteria* and *Verrucomicrobia* significantly (Figure 3D). Moreover, based on the observed microbial differences at phylum level among the three groups, the abundance of the top 30 genera with over 98% in total coverage was visualized with a heatmap (Figure 3E). In summary, compared with the other two groups, genera such as *Bacteroides, Odoribacter, Barnesiella*, were enriched, while *Allobaculum* was reduced in AOM/DSS group. Besides, compared to the AOM/DSS group, although BBR treatment decreased the relative abundance of *Bifidobacterium, Barnesiella*, and *Odoribacter*, it also reduced opportunistic pathogens (such as *f_Erysipelotrichaceae* and *Alistipes*), and increased the relative abundance of beneficial bacteria (including *Alloprevotella, Flavonifractor, Oscillibacter*, and *Parabacteroides*). These results indicated that BBR repaired the imbalance of gut microbiota partly in AOM/DSSS mice.

### Predicted Metabolic Functions for BBR Treatment Based on 16S rRNA

**FIGURE 4:**
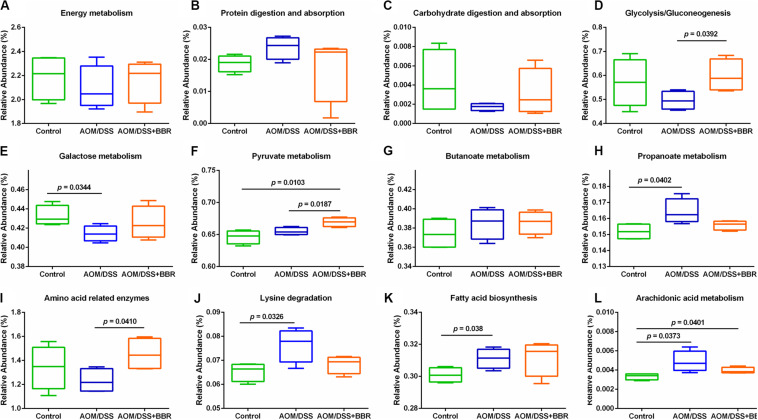
Inferred gut microbiome functions by PICRUSt from 16S rRNA gene sequences among groups. **(A–C)** Overall conditions of basic metabolism; **(D–F)** gluco-related metabolism; **(G,H)** SCFAs-related metabolism; **(I,J)** amino acid-related metabolism. **(K,L)** Lipid-related metabolism.

To predict the microbial community functions, we used PICRUSt analysis to explore the gut microbiome functions related to AOM/DSS or BBR intervention. Several metabolic pathways were changed significantly after treated with AOM/DSS and BBR. The box plots (Figures 4A–L) demonstrated that although there was no significant difference among the three groups in the overall conditions of basic metabolism, including energy metabolism, protein digestion and absorption, and carbohydrate digestion and absorption, the metabolic rate of AOM/DSS + BBR group was between control group and AOM/DSS group, suggesting that BBR intervention could reverse the metabolic abnormality caused by AOM/DSS. To be specific, functions related with glycometabolism (including glycolysis/gluconeogenesis, fructose and mannose metabolism, galactose metabolism) were decreased in the AOM/DSS group compared with other groups, while the situations were improved after BBR administration (Figures 4D–F). In addition, SCFAs-related metabolisms (propanoate metabolism and butanoate metabolism) were enhanced in AOM/DSS group compared to the control group (Figures 4G,H). Besides, amino acid-related metabolisms (Figures 4I,J) and lipid metabolism (Figures 4K,L) were changed significantly after treated with AOM/DSS and BBR. Specifically, the lysine degradation, fatty acid biosynthesis, and arachidonic acid metabolism were increased obviously in AOM/DSS induced colorectal carcinogenesis, which were partially reversed by BBR treatment. Additionally, compared to AOM/DSS group, the amino acid related enzymes were elevated remarkably in AOM/DSS + BBR group.

### Identification of Metabolites in Feces Samples

**FIGURE 5:**
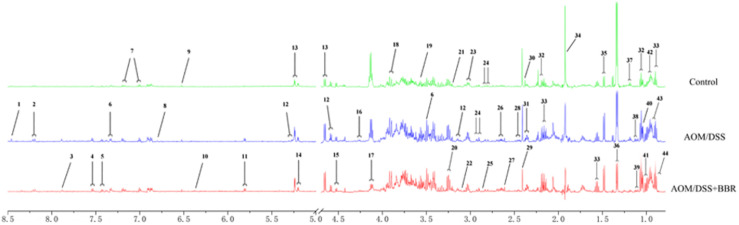
Typical 1H-NMR spectrum of fecal sample in different groups. Typical 600 MHz ^1^H NMR spectra of feces samples in the different groups. 1, formate; 2, hypoxanthine; 3, xanthine; 4, tryptophan; 5, phenylalanine; 6, 3-hydroxyphenylacetate; 7, tyrosine; 8, homovanillate; 9, fumarate; 10, urocanate; 11, uracil; 12, galactose; 13, glucose; 14, D-xylose; 15, arabinose; 16, threonine; 17, lactose; 18, aspartate; 19, glycine; 20, taurine; 21, methano; 22, choline; 23, lysine; 24, asparagine; 25, trimethylamine; 26, methionine; 27, methylamine; 28, glutamine; 29, succinate; 30, proline; 31, glutamate; 32, propionate; 33, butyrate; 34, acetate; 35, alanine; 36, lactate; 37, ethanol; 38, 3-methyl-2-oxoisovalerate; 39, 2-oxoisovalerate; 40, isobutyrate; 41, valine; 42, leucine; 43, isoleucine; 44, valerate.

The metabolites of feces were identified according to the previous studies and the HMDB and BMRB database. Forty-four feces metabolites were detected (Figure 5) and mainly focused on glucose, amino acids, SCFAs, and pyrimidines.

### Metabolic Analysis Patterns in Feces Samples

**FIGURE 6:**
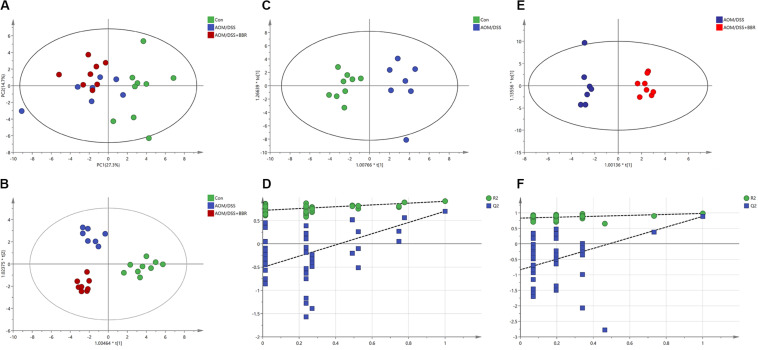
Metabolic analysis. **(A,B)** The PCA scatter plot and OPLS-DA score plots of the ^1^H NMR data of feces samples among control, AOM/DSS, and AOM/DSS + BBR group. **(C,D)** The OPLS-DA score plots and validation plot based on the ^1^H NMR data of feces samples obtained from control and AOM/DSS group. **(E,F)** The OPLS-DA score plots and validation plot based on the ^1^H NMR data of feces samples obtained from AOM/DSS and AOM/DSS + BBR group.

To elucidate the effect of AOM/DSS or BBR on fecal metabolites, the ^1^H NMR-based metabolic profiling was performed on fecal samples. The PCA scatter plot (Figure 6A) showed that the samples from the control group were obviously separated from that in other two groups, while the separation between AOM/DSS group and AOM/DSS + BBR group was not significant. However, the gathering in AOM/DSS + BBR group was closer to control group than AOM/DSS group, indicating that the composition of metabolites in AOM/DSS + BRR group have more similarity with the control group than with the AOM/DSS group. Moreover, the good separations and clustering among the three groups were displayed in the OPLS-DA score plot (Figure 6B), suggesting that there were significant differences in the fecal metabolites among these three groups. To further analyze the difference of fecal metabolites between the control and AOM/DSS groups, or AOM/DSS, and AOM/DSS + BBR groups, the score plot of OPLS-DA were carried out, and the model parameters of permutation analysis for different groups were as follows (Figures 6C–F): control vs. AOM/DSS: R2Y = 0.901, Q2 = 0.678; AOM/DSS vs. AOM/DSS + BBR: R2Y = 0.98, Q2 = 0.885. The R2 and Q2 values indicated that the models are stable and accurately predictive.

### BBR Changed Fecal Metabolites in AOM/DSS Mice

**FIGURE 7:**
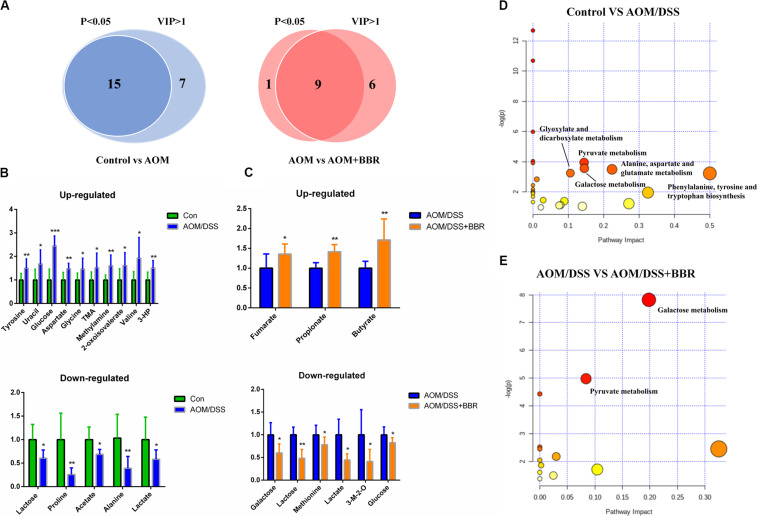
Differential metabolites and metabolic pathways. **(A)** Differential metabolites were identified based on *P* < 0.05 and VIP > 1 as the filter for control and AOM/DSS group, AOM/DSS and AOM/DSS + BBR group. **(B,C)** The change of differential metabolites in control and AOM/DSS group, AOM/DSS and AOM/DSS + BBR group. **(D,E)** Meaningful metabolic pathways in the comparison of control and AOM/DSS group, AOM/DSS and AOM/DSS + BBR group. 3-HP, 3-hydroxyphenylacetate; 3-M-2-O, 3-methyl-2-oxoisovalerate. (Control group: *n* = 9; AOM/DSS group: *n* = 7; AOM/DSS+BBR group: *n* = 8). *P < 0.05, **P < 0.01; ***P < 0.001.

To identify the significantly altered metabolites after treated with AOM/DSS or BBR, the criteria of either *P* < 0.05 at univariate statistics or VIP > 1 at multivariate statistical analysis was used. With the criteria, 15 or 22 differential metabolites were determined between control group and AOM/DSS group, and 15 of them were selected with the double criteria (Figure 7A). Similarly, 10 or 15 differential metabolites were determined between AOM/DSS group and AOM/DSS + BBR group, and 9 of them were selected with the double criteria (Figure 7A). These changes of differential fecal metabolites were showed in Figures 7B,C.

**FIGURE 8:**
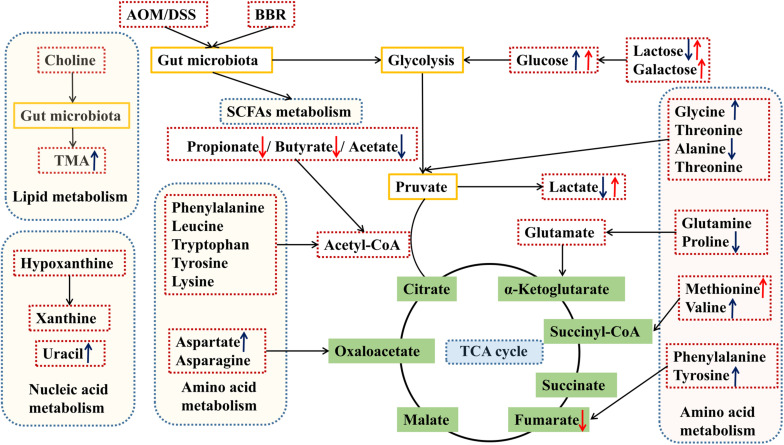
Schematic diagram of the altered pathways in feces samples in the different groups. Metabolites marked with ↑ (up-regulated) or ↓ (down-regulated) are metabolites with significant differences. Blue and red indicates AOM/DSS group vs. control group, and AOM/DSS+BBR group, respectively. TMA, trimethylamine.

Based on the above results of differential metabolites, pathway analysis was carried associated with AOM/DSS or BBR treatment intervention by using an online server, MetaboAnalyst 4.0^4^. The dominant changing pathways detected were gathered in galactose metabolism, pyruvate metabolism, amino acid metabolism (alanine, aspartate and glutamate metabolism, phenylalanine, tyrosine, and tryptophan biosynthesis), and glyoxylate and dicarboxylate metabolism. Details are showed in Figures 7D,E. Also, the levels of butyrate and propionate were increased significantly after treated with BBR, implicating that BBR increased SCFAs metabolism in AOM/DSS mice. Taken together, the changes of remarkable metabolites in metabolic pathways are further summarized in Figure 8.

### Correlation of Gut Microbiota and NMR Identified Fecal Metabolites

**FIGURE 9:**
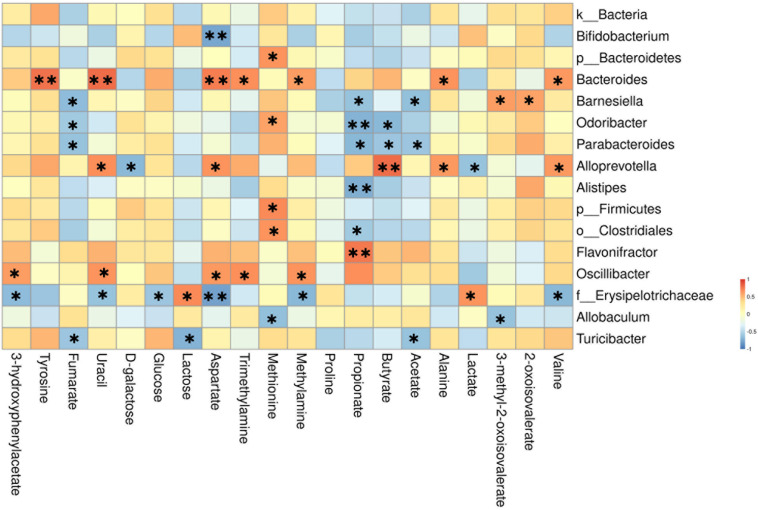
Heatmap of the correlation between the altered microbial community and significantly changed metabolites. The color indicates Spearman’s correlation coefficient, and significant correlations are noted by adjusted *P* (^∗^*P* < 0.05, ^∗∗^*P* < 0.01).

To better illustrate the association between gut microbiota and metabolic changes, we used spearman’s correlation analysis on the microbiota and metabolites that changed significantly by AOM/DSS or BBR treatment. Based on the heatmap (Figure 9), we found that *Barnesiella, Odoribacter, Parabacteroides, Alistipes, o__Clostridiales, Alloprevotella*, and *Flavonifractor* were closely correlated with the SCFAs producing, including propionate, butyrate, and acetate. Besides, *f__Erysipelotrichaceae* was closely correlated with metabolites associated with glycometabolism, including glucose, lactose, and lactate. Besides, *Bacteroides* and *Alloprevotella* (both belong to Bacteroidetes phylum) were positively correlated with the amino metabolism, including tyrosine, aspartate, alanine, and valine.

## Discussion

Berberine, as a traditional Chinese herbal medicine, has long been used to treat intestinal infections in China (31). Modern research has elucidated that BBR could regulate gut microbiota to treat obesity, collagen-induced arthritis, periodontal bone loss, and insulin resistance in animal models (26, 32–34). In addition, more recent attention has been turned to the correlation of gut microbiota and fecal metabolites in the progress of diseases, current literatures have also confirmed that metabolites played an important role in the pathological progression of CRC (35, 36). Although the study had reported that BBR restored the gut microbiota and alleviated the development of CRC in Apc min/+ mice fed with HFD, the overall effect of BBR on both microorganisms and metabolites of CRC remains unknown (27). In this study, we adapted the commonly CRC model induced by AOM and DSS, and treated with or without BBR to illustrate the inhibitory effect of BBR on CRC. The results demonstrated that BBR treatment could inhibit the development of CRC, evidenced by the decreased weight loss, reduced number of polyps, better pathological morphology, and decreased the expression of Ki-67 compared with AOM/DSS group.

Considering the anti-bacterial effect of BBR and the evidence-based study on the relationship between the gut microbiota and CRC, we planned to investigate the inhibition of BBR on CRC from the perspective of the gut microbiota, combined with detecting the metabolic alterations *in vivo*, to provide further circumstantial evidence.

### The Changed Composition of Gut Microbiota

16S rRNA sequencing is commonly used to measure the composition and structure of the gut microbiota. It is well known that decreasing diversity and richness of gut microbiota often occur in CRC (37). However, our study has a different result. Herein, we found that there is a significant difference in richness indices, but without obvious difference in diversity indices between the control group and AOM/DSS group, indicating that the number of species was lower in AOM/DSS group, but the species distribution is similar between the two groups. The decreased microbiota abundance in AOM/DSS group may be associated with the symptoms of diarrhea and hematochezia. Interestingly, no difference in diversity may be related to an increase in pathogenic bacteria or conditional pathogenic bacteria in AOM/DSS mice, such as bacteria in the *Firmicutes* (*Turicibacter, o__Clostridiales*, and *p__Firmicutes*) and *Bacteroidetes* (*Odoribacter, Bacteroides*, and *Barnesiella*) phyla. Additionally, compared to the AOM/DSS group, the richness of microbiota were decreased after treated with BBR, which partially due to the anti-bacterial effect of BBR. Studies had confirmed that BBR can effectively inhibit several pathogenic bacteria, such as *E. coli*, *Staphylococcus aureus*, and *Pseudomonas aeruginosa* (38, 39). On the contrary, BBR also can increase some beneficial bacteria (such as *Alloprevotella, Flavonifractor, Oscillibacter, and Parabacteroides*), which may be the reason why the diversity did not decrease after treated with BBR. Furthermore, we carefully examined the microbiota distribution at the different taxonomic level and found that BBR treatment decreased the relative abundance of *Actinobacteria* and *Verrucomicrobia* significantly, and the ratio of *Firmicutes/Bacteroidetes* was also decreased at the phylum level. In the aforementioned study, they found the level of *Verrucomicrobia* was increased obviously and it exhibited pro-inflammatory properties in AOM/DSS mice (37). Similarly, a reduction in the ratio of *Firmicutes/Bacteroidetes* had an effect on the prevention and treatment of CRC (40). Therefore, these results demonstrated that BBR partially restored the enteric microbiome community in AOM/DSS mice.

Additionally, previous studies have reported that different bacteria performed different mechanisms on colorectal carcinogenes, such as *f_Erysipelotrichaceae*, which can promote inflammation in the intestine (41). *Allobaculum*, which can inhibit the inflammatory response and antineoplastic properties via decreasing p-IKK and TNF-α and increasing IL-10 expression (42). Consistently, the gut microbiota of BBR treatment group mice had more *Allobaculum, Parabacteroides*, along with low *f_Erysipelotrichaceae, Alistipes*, that can form a co-occurring bacterial network to inhibit CRC (43, 44). Taken together, the altered composition of gut microbiota provided a scientific basis for research on the inhibition mechanisms of BBR for CRC.

### Gut Microbiota and Related Metabolic Pathways

Short-chain fatty acids, such as butyrate, propionate, acetate, are the main metabolites produced by microbial fermentation. Current literature has confirmed that SCFAs could inhibit the development of CRC by suppressing the inflammatory response in the intestine (45). In this study, we found a lower acetate level in feces samples in AOM/DSS group by ^1^H NMR analysis, which may be associated with the relative abundance of microbiota. As previously reported, acetate was mainly produced by the Bacteroidetes phylum (46). Consistently, the relative abundances demonstrated that the Bacteroidetes phylum was reduced in AOM/DSS group compared to the other groups. Furthermore, the concentration of SCFAs (including butyrate and propionate) were up-regulated markedly after BBR treatment in AOM/DSS mice, which may be related to the increase in the relative abundance of *Allobaculum, Alloprevotella, Flavonifractor, Oscillibacter* and reduction in *Odoribacter, Barnesiella* and *Alistipes* in AOM/DSS + BBR group (47–49). For instance, *Allobaculum*, one of the butyrate-producing bacteria, treated DSS-induced colitis by elevating the expression of butyrate in mice (50). Meanwhile, it was also found that the decrease of *Odoribacter* and *Alistipes* was associated with the increase of propionate and butyrate concentrations in the feces (51). These previous results are similar to those of microbiota and metabolic correlation analysis in our study, which further confirmed that this altered microbiota was related to SCFAs metabolism. Therefore, BBR may change the intestinal microenvironment and inhibit the expression of inflammatory factors by increasing the concentration of SCFAs, thereby to reduce intestinal mucosal damage and inhibit colorectal carcinogenesis.

Metabolic reprogramming is a characteristic of cancer (52). In this study, we found significant changes in metabolic pathways after treated with AOM/DSS by functional predictions and metabonomics analysis. These changes mainly manifested in glycometabolism (including glycolysis gluconeogenesis, galactose metabolism, and pyruvate metabolism), amino acid metabolism, and lipid metabolism. Specifically, the glycometabolism in AOM/DSS group was obviously abnormal compared to the other two groups. Metabolite analysis further revealed that the concentration of glucose increased significantly in the gut of AOMDSS group mice, which may provide enough energy for tumor growth. As we all know, tumor cells need more glucose than normal cells in the process of growth (53). It is noteworthy that BBR could improve energy metabolism by regulating bioactive metabolites of the gut microbiota (54). Hence, the abnormal glycometabolism was reversed significantly and the level of glucose in the gut was reduced dramatically in AOM/DSS mice after BBR administration, suggesting that BBR may inhibit the pathological progression of CRC by decreasing glucose content in the gut and inhibiting tumor glycometabolism. Interestingly, these metabolic changes may be owing to the BBR induced repairment of the gut microbiota disorders in AOM/DSS mice. Study has confirmed that walnut green husk polysaccharide improve glucose metabolism, lipid metabolism and decrease oxidative stress in high fat diet induced obesity mice by reversing the disorders of gut microbiota, decreased the relative abundance of *Verrucomicrobia* and increased the relative abundance of Deferribacteres at the phylum level, and enriched the relative abundance of *Allobaculum, Alloprevotella* at the genus level (55). Similarly, BBR inhibited the progression of CRC by increasing energy metabolism, decreased the relative abundance of Verrucomicrobia at the phylum level, and increased the relative abundance *Allobaculum, Alloprevotella* at the genus level. Therefore, these results confirmed that BBR inhibited CRC by regulating gut microbiota and bioactive metabolites. Additionally, the changes in amino acid and lipid metabolisms caused by BBR treatment are much smaller than changes in glycometabolism. Considering the lower richness caused by BBR treatment, fermentation of amino acids or lipids was probably affected due to the decreased amounts of related bacteria.

Moreover, it should be noted that the level of trimethylamine (TMA) was also increased significantly in AOM/DSS group, which may relate to lipid metabolism. It has been reported that the concentration of TMA was influenced by diet and gut microbiota (56). In this study, all mice were given the same food, thereby the increase of TMA in AOM/DSS group was associated with the alteration of gut microbiota. What is more, it is important that trimethylamine oxide (TMAO) is produced by the oxidation of TMA in the liver, which has been identified as a risk factor for CRC (57, 58). Currently, several lines of evidence have suggested that inflammation, oxidative stress and DNA damage may be the potential molecular mechanism to explain the link between TMAO and CRC (59). Therefore, reducing the production of TMA by modulating gut microbiota may be an effective target for the treatment of CRC. Notably, some previous studies had demonstrated that BBR could reduce TMA expression by modifying gut microbiota (60, 61). In the present study, different from the previous findings in mice, BBR did not significantly reduce the expression of TMA in the gut of AOM/DSS mice. The difference between previous reports and our results might reflect the variable microbiota profiles influenced by multiple factors, including diet, gender, age, and environment. Although no difference in the expression of TMA after treated with BBR in this study, based on the regulatory effect of BBR on gut microbiota and previous studies, it is still a worthy target to explore the inhibitory of BBR on TMA and TMAO in the treatment of CRC.

## Conclusion

The present study demonstrates that BBR administration can effectively reduce intestinal tumor development with lower macroscopic polyps and ki-67 expression of intestinal tissue in mice. We also reveal that the altered gut microbial composition with BBR treatment was marked by a decrease of pathogenic bacteria and an increase of beneficial bacteria compared with AOM/DSS group. After combining the predicted metabolic functions of microbiota with the analysis of ^1^H NMR detected fecal metabolites, we focused on three types of metabolites and pathways in which microbiota are indispensably involved. The study found that BBR treatment may inhibit the pathological progression of CRC by increasing the rates of some microbiota-mediated energy metabolisms (especially glycometabolism), which can directly influence the content of fecal metabolites and indirectly regulate tumor metabolism. Also, the alterations in SCFAs and TMA levels achieved high consistency with the alterations of gut microbiota, suggesting that microbiota and metabolites play an important role in the BBR treatment of CRC. Furthermore, in-depth study with larger sample size is need to further clarify the clear mechanisms of BBR inhibiting CRC by regulating the microbiota and metabolites.

## Data Availability Statement

The 16S DNA datasets generated for this study can be found in the NCBI-PRJNA659636, the BioSample accessions are SRR12649436 to SRR12649447.

## Ethics Statement

The animal study was reviewed and approved by the Experimental Animal Ethical Committee of the Zhejiang Chinese Medical University.

## Author Contributions

HC, FZ, JZ, QY, and YG conceived the study design, supervised the scientific work, analyzed the data, and prepared the figures and table. HC, FZ, JZ, and XZ performed the experiments. HC, FZ, and JZ wrote the manuscript. QY and YG revised the manuscript. All authors contributed to and approved the final manuscript.

## Conflict of Interest

The authors declare that the research was conducted in the absence of any commercial or financial relationships that could be construed as a potential conflict of interest.

## Footnotes

1 http://www.hmdb.ca/

2 http://www.bmrb.wisc.edu/metabolomics

3 https://software.broadinstitute.org/morpheus/

4 http://www.metaboanalyst.ca
